# Prevalence of first sexual intercourse before age 14 and forced sexual intercourse in the 2004 Pelotas Birth Cohort

**DOI:** 10.1016/j.jped.2026.101582

**Published:** 2026-07-22

**Authors:** Mariangela Freitas da Silveira, Isabel Oliveira Bierhals, Luciana Tovo-Rodrigues, Alicia Matijasevich, Iná S. Santos

**Affiliations:** a Universidade Federal de Pelotas, Post-graduate Program in Epidemiology, Pelotas, RS, Brazil; b Universidade do Extremo Sul Catarinense, Postgraduate Program in Population Health, Criciúma, SC, Brazil; c Universidade de São Paulo (USP), Faculdade de Medicina (FM), Department of Preventive Medicine, São Paulo, SP, Brazil

**Keywords:** Adolescent, Sexual intercourse, Sexual behavior, Sexual abuse, Sex education

## Abstract

**Objective:**

The study described early sexual initiation, forced sexual initiation, and associated factors in young participants of a Brazilian birth cohort.

**Methods:**

Participants who, at the 18-year-of-age follow-up, answered yes to the question “Have you ever had sexual intercourse (had sex)?” were included. They were asked about age at first intercourse (< 14 or ≥ 14) and whether it was consensual (“because I wanted to” or “because I was forced”). Factors associated with these outcomes were analyzed by Poisson regression with robust variance according to a hierarchical model.

**Results:**

The sample consisted of 3255 participants, with a prevalence of sexual intercourse before 14 years of age of 13.3% and of forced first sexual intercourse of 1.6%. First intercourse before 14 years of age was more likely to occur in boys, those who identified as asexual or in the category “other” when asked about their sexual orientation, who currently used drugs daily/almost daily, whose first sexual intercourse was forced, and who had more lifetime sexual partners. First forced sexual intercourse was more likely to occur in girls, those who identified as pansexual, who had sexual intercourse before 14 years of age, and who had more lifetime sexual partners.

**Conclusions:**

Better guidance is recommended for adolescents regarding sexual abuse and violence and methods of self-preservation and reporting.

## Introduction

For Brazilian law [1], sexual intercourse or sexual act with a person under 14 years of age, regardless of the victim’s consent, previous sexual experience, or existence of a romantic relationship with the offender, is considered rape of a vulnerable person. Authors suggest that sexual initiation prior to 15 years of age should be considered precocious, while others propose ages varying from 13 to 18 years. Several authors have pointed out that the younger the age of sexual initiation, the higher the likelihood of sexually transmitted infections (STIs), HIV, unplanned teenage pregnancy, maternal mortality, cervical cancer, harmful consumption of alcohol and drugs, and dropping out of or falling behind in school [2].

A review by the World Health Organization (WHO) estimated the global prevalence of childhood sexual victimization at 27% among girls and 14% among boys. Other studies reported this prevalence among girls around 7–8%, being more common in girls than boys [3].

In Brazil, the average age at first sexual intercourse was 13.7 for girls and 12.9 for boys in 2015, being later for middle-class adolescent girls (17 years) than for lower-class girls (15.7 years) [4,5]. A review of Brazilian studies found that sexual debut of boys and girls occurred between 14 and 17 years of age [6], with boys starting earlier than girls. Factors that could postpone the onset of sexual intercourse include religious practice, higher education, being enrolled in school, having more educated parents and living in more rural areas. Associated with earlier sexual intercourse are use of tobacco, alcohol, and illicit drugs [6].

A 2002 Brazilian adolescents’ study found that 52% had already sexually debuted, with a median age at first sexual intercourse of 16.7 years [7]. In 8.4%, sexual initiation occurred before 14 years. Factors associated with earlier sexual debut were male sex, not living with either parent, unemployment, no religious practice, alcohol, tobacco, or drug consumption in the last month [7]. In a 2004–2005 study of 1145 adolescents up to 15 years, 11.6% had already had sexual intercourse, 5.2% under 14 years of age at the time. A 2007–2008 population-based study in Pelotas, with 18–24-year-olds, found that 8.8% sexually debuted before the age of 13 and 24.6% by 14 years of age. Directly associated with early sexual initiation were male sex, low socioeconomic level, low education, separated parents, living with a partner, no religious practice, tobacco and drug use [8].

WHO defined sexual violence as: “Any sexual act, attempt to obtain a sexual act, unwanted sexual comments or advances, or acts to traffic or otherwise directed against a person’s sexuality using coercion, by any person regardless of their relationship to the victim, in any setting, including but not limited to home and work” [9]. The younger the age of first sexual intercourse, the higher the likelihood of coercion. A WHO 2015 multi-country study found a prevalence of forced first sexual intercourse for women from < 1% in Japan to nearly 30% in rural Bangladesh [10]. Surveys asking women about ‘unwanted’ sexual initiation typically find rates much higher than when they ask about ‘forced’ initiation [3].

A study of 13,310 American women, aged 18–44 years, found a 6.5% prevalence of forced sexual debut, with the mean age being 15.6 years and 17.4 years for unwanted and voluntary sexual debut, respectively. Women with a forced sexual debut were more likely to have an unwanted first pregnancy and abortion, and to report illicit drug use [11].

In urban Brazil, sexual abuse before 15 years of age was 6% in face-to-face interviews and 9% in anonymous reports, with 8% and 12% in the provincial setting, respectively. Prevalence of forced first sexual intercourse was 3% (urban) and 4% (provincial), increasing to 14% (urban) and 11% (provincial) if it occurred before 15 years of age [12].

Sexual violence against men is a very sensitive and neglected area of investigation, and is associated with perpetration in later life; this subject must be addressed to prevent subsequent sexual violence [12].

In studies from 2007–2028, a prevalence of forced sexual initiation among adolescent girls and young women, ranged from 14.7% (Zimbabwe) to 38.9% (Malawi), being higher among those aged 13–15 years [13]. A Central American study on child sexual abuse before 15 years of age found rates of 4.7% in Guatemala and 7.8% in Honduras [14]. In other studies, 20% of women and 5%−10% of men reported having been victims of sexual violence in childhood. Factors associated included: low education levels, child abuse, family violence, harmful alcohol use, and multiple partners [10].

The Institute of Applied Economic Research (IPEA) reported, between 2009 and 2019, 63,309 cases of rape in children (0–10 years) and 98,221 in young people (11 to 20 years) [15]. A study of 996 university students found a 12.1% lifetime prevalence of forced sexual intercourse. Associated factors included female sex, non-heterosexual sexual orientation, sexual intercourse before 14 years of age, and domestic violence in childhood [16]. Forced sexual intercourse increased the risk of STIs and suicide [16].

Using data from the 2004 Pelotas Birth Cohort, this study aims to characterize sexual intercourse before 14 years of age and first forced sexual intercourse.

## Methods

### Sample

The 2004 Pelotas Birth Cohort is a longitudinal population-based study of children born to mothers residing in the Pelotas urban area [17,18]. From January 1 to December 31, 2004, all live births were eligible for participation, with mothers of 4231 newborns agreeing to participate. Cohort members were followed up at 3, 12, 24, and 48 months and at 6, 11, 15, and 18 years of age [[17], [18], [19]]. The follow-up rate at 18 years of age was 85%.

The present study included participants who provided data on sexual activity at the 18-year follow-up through a confidential self-administered questionnaire. Data from the perinatal study and 6-year follow-up were also used.

### Outcomes

In the self-administered questionnaire, participants who responded affirmatively to the question “Have you ever had sexual intercourse (had sex)?” were asked about age at first sexual intercourse and whether it was consensual through the following questions: “How old were you [exactly] at your first intercourse?”, and “Your first intercourse occurred: (a) because I wanted to, or (b) because I was forced to”. For the analysis, age at first sexual intercourse was dichotomized as < 14 or ≥ 14 years.

### Covariates

Perinatal characteristics were used as independent variables: family income (quintiles), maternal age (< 18, 18–21, 22–34 and > 34 gt; 34 years), maternal education (0–4, 5–8, 9–11, and ≥ 12), and participant sex. From the 6-year follow-up, the variables “maternal religious practice (no, yes)” and “race of the cohort participant (Black, Mixed, White, or other)” were used.

At the 18-year follow-up, overweight was calculated based on the weight and height measures. Data on all other variables were collected through a confidential self-administered questionnaire. BMI was classified according to the 2006–2007 WHO reference values for 0 to 19 years old [20]. Those with a z-score > 1 were considered overweight [21]. The current smoking variable was derived from the following questions: “Have you ever habitually smoked at least once a week?” and “Do you still smoke? (yes or no)”. Alcohol consumption was investigated using the Alcohol Use Disorders Identification Test [22], subdivided into the following consumption patterns: 0 to 7 (low-risk consumption), 8–15 (risky consumption), 16–19 (harmful or high-risk consumption) and ≥ 20 (possible dependence) [23]. For the analysis, “harmful or high-risk use” and “possible dependence” were merged. Regarding illicit drug consumption, the participants were asked about the following substances: marijuana, pills “to sleep or stay calm”, snorted cocaine, injected cocaine, heroin, pills “to get high or turned on”, chloroethane spray (*loló*), ecstasy, marijuana laced with crack (*pitico*), LSD, crack, rubber cement, and other. The response options for each substance were: “I’ve never used it”, “I only tried it once”, “I used to use it, but I don’t anymore”, “I use it from time to time”, “I only use it on weekends”, and “I use it every day or almost every day”. For the analysis, the responses were grouped into 4 categories: “I have never used/Tried it once”, “I have used it, but I no longer use it”, “I use it occasionally or only use on weekends” and “I use it every day or almost every day.” Sexual orientation was assessed with the question “What is your sexual orientation? Mark the one you consider predominant”, which included the following response options “heterosexual: I am attracted to individuals of the opposite sex”, “homosexual: I am attracted to individuals of the same sex”, “bisexual: I am attracted to both sexes”, “asexual: I am not attracted to either sex”, “pansexual: I am attracted to people regardless of sex”, or “other”. “Asexual” and “other” were merged for the analysis. Immunization for human papillomavirus was assessed using the question “Have you been vaccinated against human papillomavirus? (responses: no, yes, or I don’t know)”. The number of lifetime sexual partners was determined with the question “How many people have you had sex with in your life?”, and the responses were categorized as 1, 2, 3–9 and ≥ 10.

### Statistical analysis

Collected data were entered directly into Pendragon [24] and REDCap software [25]. Analysis were performed in Stata 16.0 (StataCorp, LLC, College Station, TX, USA). Descriptive analysis included absolute and relative frequencies of the variables. The chi-square test of heterogeneity was used to compare the birth characteristics of the included participants with the overall cohort. Prevalence of sexual intercourse, sexual intercourse before 14 years of age, and first forced sexual intercourse were described for the entire cohort and stratified by sex.

To evaluate factors associated with first sexual intercourse before 14 years of age and the first forced sexual intercourse, Poisson regression with robust variance was performed, resulting in crude and adjusted prevalence ratios (aPR) with 95% confidence intervals (95%CI). Variables were included in the model according to hierarchical level and were adjusted for other variables on the same level and for those on higher levels. For both outcomes, the following hierarchical levels were used: level 1- sex, sexual orientation, and race; level 2 - family income and maternal education; and level 3 - maternal religious practice and other contemporary variables, including overweight, human papillomavirus immunization, number of lifetime sexual partners, tobacco, alcohol, and illicit drug consumption. For the outcome first sexual intercourse before 14 years, first forced sexual intercourse was included as an independent variable in level 3; for the outcome forced first sexual intercourse, first sexual intercourse before 14 years was included as an independent variable in level 3. Complementary analyses were run after stratifying the sample by sex. Only variables with p ≤ 0.20 were retained in the final model. The significance level used in the two-tailed test was p < 0.05.

### Study ethics

The 18-year follow-up of the 2004 Pelotas Birth Cohort protocol was approved by the Research Ethics Committee of the Federal University of Pelotas School of Medicine (decision 5210,484; certificate 54362821.0.0000.5317). For each follow-up investigation, written informed consent was obtained from the participant’s mother or legal guardian. At 18 years of age, the participants also provided written informed consent.

## Results

A total of 3255 participants were included in the analysis. The analyzed sample was compared to the overall cohort (N = 4231) (Table 1) and to those followed-up at 18 years of age (Supplementary Table 1). Distribution of maternal variables and perinatal and contemporary characteristics in the sample analyzed was similar to those in the overall cohort and to all those followed up at 18 years.

**Table 1:** Description of the overall 2004 pelotas birth cohort and the sample included in this analysis.

Variable	Original cohort (N = 4231)		Included in this analysis (N = 3255)		p-value^a^
	N	%	N	%	
**Maternal perinatal characteristics**					
**Family income quintile**					0.423
1	871	20.6	620	19.1	
2	854	20.2	647	19.9	
3	816	19.3	649	19.9	
4	858	20.3	698	21.4	
5	830	19.6	641	19.7	
**Maternal age at birth**					0.577
< 18	395	9.4	303	9.3	
18–21	871	20.6	632	19.4	
22–34	2398	56.7	1864	57.3	
> 34	563	13.3	455	14.0	
**Maternal education at birth (in years)**					0.121
0–4	654	15.6	457	14.2	
5–8	1731	41.4	1313	40.8	
9–11	1381	33.0	1139	35.3	
≥12	420	10.0	314	9.7	
**Maternal religious practice**					0.980
No	1949	53.4	1625	53.4	
Yes	1702	46.6	1416	46.6	
**Participant characteristics**					
**Sex**					0.294
Male	2195	51.9	1648	50.6	
Female	2036	48.1	1607	49.4	
**Race**					0.939
White	2726	68.2	2185	68.1	
Mixed, Black, or other	1272	31.8	1024	31.9	

a p-value refers to the chi-square test of heterogeneity. Source: Own authors.

**Figure 1:**
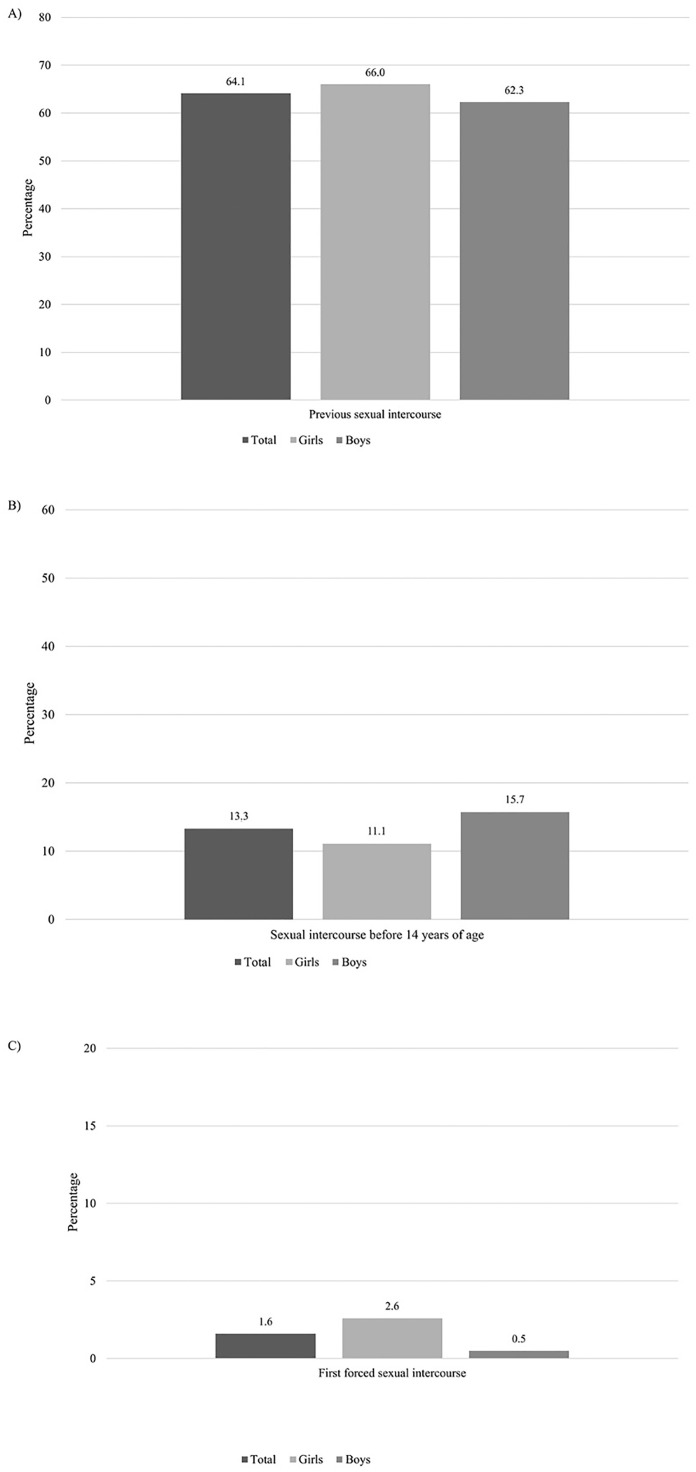
Prevalence of (A) previous sexual intercourse, (B) sexual intercourse before 14 years of age, and (C) first forced sexual intercourse, stratified by sex.

Figure 1-A shows the prevalence of previous sexual intercourse, age at first sexual intercourse (Figure 1-B), and whether the first sexual intercourse was consensual (Figure 1-C). At 18 years of age, around 64% of the sample reported previous sexual intercourse, with higher prevalence among girls (p = 0.030). The sexual debut of almost 16% of the boys and 11% of the girls occurred before 14 years of age (p = 0.002). The non-consensual sexual initiation was approximately 5 times higher in girls (2.6%) than in boys (0.5%) (p < 0.001).

Table 2 shows results of crude and adjusted analysis of factors associated with sexual intercourse before 14 years of age. In the adjusted analysis, sex remained associated with early sexual activity, with boys having a 50% higher probability of sexual intercourse before 14 years of age than girls (aPR = 1.50; 95% CI, 1.18–1.91; p = 0.001). The probability of sexual intercourse before 14 years of age was 90% higher among adolescents who identified themselves as “asexual/other” than among heterosexuals. Current drug use remained associated with the outcome, especially daily or almost daily use (aPR = 2.11; 95% CI, 1.48–3.02; p < 0.001). The association between first forced sexual intercourse and sexual intercourse before 14 years of age also remained significant in the adjusted analysis (aPR = 2.52; 95% CI, 1.30–4.88; p = 0.006). When stratifying by sex, the association between the first forced sexual intercourse and previous sexual activity was only significant in girls, with those reporting first forced sexual intercourse being 3.4 times more likely to have had sexual intercourse before 14 years of age than those who did not report being forced (95% CI, 1.91–5.85; p < 0.001). Regarding lifetime sexual partners, the higher the number, the greater the probability of sexual intercourse at < 14 lt; 14 years of age.

**Table 2:** Crude and adjusted analysis of factors associated with first sexual intercourse before 14 years of age, 2004 Pelotas Birth Cohort.

Level	Variable	Crude analysis		Adjusted analysis	
		PR (95% CI)	p-value	PR (95% CI)	p-value
**1**	**Sex**		**0.002**		**0.001**
	Male	1.42 (1.14–1.77)		1.50 (1.18–1.91)	
	Female	Ref.		Ref.	
**1**	**Sexual orientation**		0.129		**0.035**
	Heterosexual	Ref.		Ref.	
	Homosexual	0.86 (0.42–1.75)		0.82 (0.38–1.76)	
	Bisexual	0.94 (0.69–1.29)		1.16 (0.83–1.62)	
	Pansexual	1.47 (0.85–2.54)		1.70 (0.98–2.94)	
	Asexual or other	1.74 (1.06–2.85)		1.90 (1.17–3.07)	
**1**	**Race**		0.444		0.383
	White	Ref.		Ref.	
	Mixed, Black, or other	1.09 (0.87–1.38)		1.11 (0.88–1.40)	
**2**	**Family income quintile at birth**		**0.004**		0.095
	1	1.77 (1.20–2.60)		1.48 (0.97–2.27)	
	2	1.74 (1.18–2.55)		1.44 (0.93–2.21)	
	3	1.13 (0.74–1.73)		0.99 (0.63–1.55)	
	4	1.27 (0.84–1.93)		1.13 (0.72–1.75)	
	5	Ref.		Ref.	
**2**	**Maternal education at birth (in years)**		**0.004**		0.174
	0–4	2.56 (1.38–4.75)		2.05 (1.05–3.99)	
	5–8	2.12 (1.17–3.84)		1.83 (0.97–3.45)	
	9–11	1.67 (0.91–3.07)		1.62 (0.86–3.05)	
	≥ 12	Ref.		Ref.	
**3**	**Maternal religious practice**		0.624		0.400
	No	1.06 (0.84–1.34)		1.11 (0.87–1.42)	
	Yes	Ref.		Ref.	
**3**	**Overweight**		0.844		0.429
	No	Ref.		Ref.	
	Yes	1.03 (0.80–1.32)		1.14 (0.82–1.59)	
**3**	**Immunized for human papillomavirus**		0.502		0.438
	No	Ref.		Ref.	
	Yes	0.92 (0.72–1.19)		1.13 (0.79–1.61)	
	Unknown	0.82 (0.59–1.14)		0.84 (0.53–1.33)	
**3**	**Current smoking**		**<0.001**		0.861
	No	Ref.		Ref.	
	Yes	2.42 (1.90–3.08)		1.04 (0.68–1.59)	
**3**	**Current alcohol consumption**		**<0.001**		0.257
	Low-risk consumption	Ref.			
	Risky consumption	1.64 (1.23–2.18)		1.00 (0.71–1.40)	
	Harmful or high-risk use/Possible addiction	3.10 (2.20–4.38)		1.38 (0.88–2.17)	
**3**	**Current drug use**		**<0.001**		**0.001**
	Never used/Tried it once	Ref.			
	Used it, but no longer uses it	2.01 (1.47–2.75)		1.27 (0.90–1.80)	
	Use it occasionally/on weekends	2.14 (1.61–2.85)		1.28 (0.92–1.79)	
	Use every day or almost every day	3.82 (2.82–5.17)		2.11 (1.48–3.02)	
**3**	**First forced sexual intercourse**		**<0.001**		**0.006**
	No	Ref.		Ref.	
	Yes	2.74 (1.68–4.46)		2.52 (1.30–4.88)	
**3**	**Number of lifetime sexual partners**				**<0.001**
	1	Ref.		Ref.	
	2	2.25 (1.29–3.94)		2.10 (1.20–3.66)	
	3–9	4.80 (3.08–7.49)		4.30 (2.70–6.85)	
	≥10	10.79 (6.88–16.91)		8.79 (5.34–14.48)	

PR, prevalence ratio.

**Level 1:** adjusted for sex and sexual orientation.

**Level 2:** adjusted for sex, sexual orientation, family income and maternal education.

**Level 3:** adjusted for sex, sexual orientation, family income, maternal education, current drug use, first forced sexual intercourse and number of lifetime sexual partners.

Table 3 shows results of crude and adjusted analyses for first forced sexual intercourse. Female sex was a risk factor (aPR = 5.21; 95% CI, 1.97–13.81; p = 0.001). The aPR of first forced sexual intercourse was almost 5 times higher among adolescents who declared themselves pansexual (aPR = 4.86; 95% CI, 1.84–12.84; p = 0.008) than those identified as heterosexual. No association was found between first forced sexual intercourse and race, family income, maternal education, maternal religious practice, overweight, human papillomavirus vaccination, or tobacco/alcohol/drug use. Among those whose first sexual intercourse occurred before 14 years of age, the probability that it was forced was 3 times higher than among those whose first intercourse occurred at ≥ 14 years of age (aPR = 3.27; 95% CI, 1.31–8.17; p = 0.011). When stratified by sex, the probability that it was forced was approximately 4 times higher among girls whose first sexual intercourse occurred before 14 years of age than among those whose first intercourse occurred at ≥ 14 years of age (PR = 4.30; 95% CI, 1.96–9.43; p < 0.001). This association was not observed among boys, although a higher number of lifetime sexual partners was also associated with a higher prevalence of first forced sexual intercourse.

**Table 3:** Crude and adjusted analysis of factors associated with first forced sexual intercourse, 2004 Pelotas Birth Cohort.

Level	Variable	Crude analysis		Adjusted analysis	
		PR (95% CI)	p-value	PR (95% CI)	p-value
**1**	**Sex**		**0.001**		**0.001**
	Male	Ref.		Ref.	
	Female	5.22 (2.02–13.50)		5.21 (1.97–13.81)	
**1**	**Sexual orientation**		**0.002**		**0.008**
	Heterosexual	Ref.		Ref.	
	Homosexual	1.32 (0.18–9.70)		1.02 (0.14–7.40)	
	Bisexual	1.16 (0.44–3.10)		0.66 (0.25–1.79)	
	Pansexual	6.83 (2.64–17.65)		4.86 (1.84–12.84)	
	Asexual or other	2.88 (0.69–12.06)		2.28 (0.55–9.34)	
**1**	**Race**		0.407		0.260
	White	Ref.		Ref.	
	Mixed, Black, or other	1.34 (0.67–2.71)		1.48 (0.75–2.95)	
**2**	**Family income quintile at birth**		0.324		0.415
	1	2.86 (0.60–13.67)		2.56 (0.49–13.50)	
	2	1.95 (0.38–10.02)		1.87 (0.35–9.92)	
	3	3.35 (0.72–15.68)		3.15 (0.67–14.79)	
	4	4.22 (0.93–19.13)		3.67 (0.82–16.47)	
	5	Ref.		Ref.	
**2**	**Maternal education at birth (in years)**		0.916		0.918
	0–4	1.01 (0.25–3.97)		0.95 (0.23–3.93)	
	5–8	0.78 (0.22–2.70)		0.80 (0.23–2.79)	
	9–11	0.74 (0.20–2.69)		0.70 (0.19–2.55)	
	≥ 12	Ref.		Ref.	
**3**	**Maternal religious practice**		0.353		0.194
	No	1.41 (0.68–2.90)		0.60 (0.28–1.30)	
	Yes	Ref.		Ref.	
**3**	**Overweight**		0.997		0.763
	No	Ref.		Ref.	
	Yes	1.00 (0.47–2.13)		0.84 (0.27–2.64)	
**3**	**Immunized for human papillomavirus**		0.457		0.190
	No	Ref.		Ref.	
	Yes	1.79 (0.72–4.46)		0.94 (0.38–2.36)	
	Unknown	1.56 (0.53–4.60)		2.25 (0.76–6.65)	
**3**	**Current smoking**		0.637		0.384
	No	1.33 (0.41–4.33)		1.85 (0.46–7.42)	
	Yes	Ref.		Ref.	
**3**	**Current alcohol consumption**		0.461		0.630
	Low-risk consumption	Ref.			
	Risky consumption	1.73 (0.71–4.23)		1.64 (0.58–4.58)	
	Harmful or high-risk use/Possible addiction	1.64 (0.36–7.40)		1.48 (0.30–7.29)	
**3**	**Current drug use**		0.269		0.537
	Never used/Tried it once	Ref.			
	Used it, but no longer uses it	1.82 (0.69–4.83)		1.33 (0.40–4.44)	
	Use it occasionally/on weekends	2.14 (0.91–5.03)		2.00 (0.78–5.09)	
	Use every day or almost every day	0.79 (0.11–5.86)		1.17 (0.12–11.15)	
**3**	**Sexual initiation before age 14**		**0.001**		**0.011**
	No	Ref.		Ref.	
	Yes	3.58 (1.74–7.40)		3.27 (1.31–8.17)	
**3**	**Number of sexual partners in life**		**0.008**		**0.016**
	1	Ref.		Ref.	
	2	7.28 (2.04–25.93)		5.88 (1.76–19.65)	
	3–9	4.94 (1.45–16.90)		4.52 (1.35–15.16)	
	≥10	1.04 (0.11–9.94)		0.96 (0.08–11.46)	

PR, prevalence ratio.

**Level 1 and 2:** adjusted for sex and sexual orientation.

**Level 3:** adjusted for sex, sexual orientation, maternal religion, human papillomavirus vaccination, sexual intercourse before 14 years of age, and number of lifetime sexual partners.

## Discussion

At 18 years of age, approximately 64% of our sample were already sexually active, especially girls. Mean age at first sexual intercourse was 16.0 years, 15.0 years for boys and 17.0 years for girls. Mean age in the present study was higher than in a 2015 study of Brazilian adolescents (12.9 and 13.7 years for boys and girls, respectively) but was consistent with a review of Brazilian studies, which also found that boys started earlier than girls [6].

Almost 16% of boys and 11% of girls had sexual intercourse before 14 years of age. A study comparing 50 countries found that the highest prevalence of early sexual initiation was in the Americas (18.4%) and the lowest was in Southeast Asia (5.3%) [26]. A Brazilian study found that 15.4% of respondents aged 13–14 reported previous sexual intercourse, compatible with our findings. [27] Other studies from Pelotas in 2002, 2005, and 2008 found that 8.4%, 5.2%, and 8.8%, respectively, of the participants reported sexual intercourse before 14 years of age, lower rates than the present study. Variations may be due to cultural and temporal factors. A study of 8641 women found that younger women reported having sexual intercourse at a younger age than older women, which corroborates the earlier onset of sexual activity found in more recent studies [28].

Although the law does not contemplate consensual sexual practices until the age of 13, the authors asked about the participant’s own perceptions about their first sexual experience. First forced sexual intercourse was reported by 0.5% of boys and 2.6% of girls. Among women, this prevalence varies from 1% to 30%, depending on the country [10]. A Peruvian study found that the prevalence of first forced sexual intercourse was 4 times higher among women than men [3]. A study of American women aged 18 to 44 years found that the prevalence of forced sexual initiation was 6.5%, higher than in our study [11].

In our sample, boys were 50% more likely than girls to have sexual intercourse before 14 years of age. Studies have reported that boys may lie about their age at first sexual intercourse to prove their masculinity, while girls may lie out of fear, shame, or taboos. This may have occurred in our sample despite the use of a self-administered questionnaire and guaranteeing data anonymity. Several studies have reported that the prevalence of early sexual initiation differs according to sex, suggesting a widespread encouragement, across cultures, regions, and ethnicities, for boys to have sex early, and a cultural double standard that rewards boys for engaging in sexual activity while shaming girls for doing so [26].

Adolescents whose self-reported sexual orientation was “asexual” or “other” were 90% more likely to have had sexual intercourse before 14 years of age than their heterosexual peers. Men and women who identify as homosexual/bisexual tend to have an earlier sexual debut than heterosexuals. In an American study, the sexual debut of approximately 3.0% occurred at < 13 lt; 13 years. Approximately 2.4%, 1.8%, and 0.9% of respondents who reported having sex at < 13 lt; 13 years of age were men who had sex with men, bisexual, or women who had sex with women. However, of respondents reporting age at sexual debut ≥ 18 years, only 0.5% were men who had sex with men or bisexual, and 0.4% were women who had sex with women [29].

There was a strong association between female sex and first forced sexual intercourse. This prevalence varied from 1% to 30% in other studies, depending on the context [10], as well as the manner in which the question was asked, since positive response rates for “unwanted sexual initiation” were typically higher than for “forced initiation” [10].

In our sample, the association between first forced sexual intercourse and sexual intercourse before 14 years of age also remained significant, especially for girls. A study of undergraduate university students in southern Brazil also found an association between forced intercourse, female sex, and sexual intercourse before 14 years of age [16].

Those who identified as pansexual were almost 5 times more likely to have had first forced sexual intercourse than those who identified as heterosexual. Other studies have also found a higher prevalence of forced sexual intercourse among non-heterosexuals. In our sample, first sexual intercourse before 14 years of age increased by 3 times the likelihood that it was forced. Regarding lifetime sexual partners, the higher the number, the higher the probability of sexual intercourse before 14 years of age. Other studies have also found an association between sexual violence and multiple partners [10,30].

Sexual initiation at a younger age remains a public health concern because it is related to negative health outcomes in adolescence and young adulthood, such as unsafe sex, unintended pregnancy, STIs, mental health problems, substance use, eating disorders, low self-esteem, antisocial personality, depression, suicidal ideation, and suicide attempts [31]. Early sexual debut is also related to multiple sexual partners, unsafe abortion, physical aggression, and poor school performance [31]. The relationship between age of sexual initiation and substance use is largely explained as a consequence of sexual behavior [32]. Sexual violence increases the risk of STIs and suicide risk [16], unwanted first pregnancy or abortion. Victims of forced sexual initiation more frequently report illicit drug use and fair or poor health [11].

The authors conclude that determining the prevalence of sexual intercourse before 14 years of age and first forced sexual intercourse is an important finding in our population, especially for girls. Sexual initiation and sexual violence should be topics covered in school curricula and in adolescent consultations in health services. Since sexual victimization, especially during childhood, is associated with perpetration in later life, this subject should receive special attention to prevent subsequent sexual violence [12].

Our study’s strengths include being a population-based cohort study, with access to reliable data and multiple follow-ups. The large number of participants and small percentage of losses contribute to the sample’s representativeness. Moreover, collecting behavioral data through confidential self-administered questionnaires reduces the risk of information bias.

Study limitations include the fact that behavioral variables were collected at 18 years, which could have led to reverse causality bias regarding the outcome variables. The low reported prevalence of forced sexual intercourse is a statistical limitation, reducing the power of the study to detect statistically significant associations. Other relevant information was unavailable, such as the identity of the first sexual partner and/or the perpetrator of the first forced sexual intercourse, as well as contraceptive use.

Sexual intercourse before 14 years of age is 1.5 times more common among boys than girls, while girls are 5 times more likely to be victims of first forced sexual intercourse. There is a strong relationship between sexual intercourse before 14 years of age and first forced sexual intercourse among girls, demonstrating the vulnerability of young girls to sexual abuse. The results support the recommendation of better guidance for adolescents about sexual initiation, abuse and violence, and methods of self-preservation and reporting. Health and education professionals should be trained to identify cases. Studies that better clarify circumstances of abuse and rape are needed to improve primary prevention, including school programs.

## Funding

This article is based on data from the study "Pelotas Birth Cohort, 2004" conducted by the Postgraduate Program in Epidemiology at Universidade Federal de Pelotas, with the collaboration of the Brazilian Public Health Association (ABRASCO). From 2009 to 2013, the Wellcome Trust supported the 2004 birth cohort study. The World Health Organization, National Support Program for Centers of Excellence (PRONEX), Brazilian National Research Council (CNPq), Brazilian Ministry of Health, and Children’s Pastorate supported previous phases of the study. This article is based on data from the study "Pelotas Birth Cohort, 2004" conducted by the Postgraduate Program in Epidemiology at Universidade Federal de Pelotas, with the collaboration of the Brazilian Public Health Association (ABRASCO). From 2009 to 2013, the Wellcome Trust supported the 2004 birth cohort study. The World Health Organization, National Support Program for Centers of Excellence (PRONEX), Brazilian National Research Council (CNPq), Brazilian Ministry of Health, and Children’s Pastorate supported previous phases of the study. The 11-year follow-up was supported by the Department of Science and Technology (DECIT) of the Brazilian Ministry of Health, CNPq and the Research Support Foundation of the State of São Paulo (FAPESP). The 15-year follow-up was funded by DECIT, CNPq, the Research Support Foundation of the State of Rio Grande do Sul (FAPERGS), FAPESP, and the L’Oréal-Unesco-ABC Program for Women in Science in Brazil-2020. The 18-year follow-up was supported by DECIT, CNPq, FAPERGS, L’Oréal-Unesco-ABC Program for Women in Science in Brazil-2020, and by the All for Health Institute. MFS, LTR, AM and ISS hold a Research Productivity Fellowship from the Brazilian National Council for Scientific and Technological Development (CNPq).

## Data availability

The data that support the findings of this study are available from the corresponding author.

## Conflicts of interest

The authors declare no conflicts of interest.

## Appendix Supplementary materials

Image, application 1
