# Supplementary material for: Prevalence of first sexual intercourse before age 14 and forced sexual intercourse in the 2004 Pelotas Birth Cohort
Source: J Pediatr (Rio J). 2026 Jul 22;102(5):101582. doi: 10.1016/j.jped.2026.101582 (PMC13396709; doi:10.1016/j.jped.2026.101582)
Supplement: Supplementary file 1 [file mmc1.docx]

**JPED-D-26-00060_Suplementary Material**

**Supplementary Table 1** Description of the 2004 Pelotas Birth Cohort at 18 years of age and the participants who provided information about sexual activity.

| **Variable** | **Cohort at 18 years of age**  **(N = 3595)** | | **Included in the analysis**  **(N = 3255)** | | **p-value** |
| --- | --- | --- | --- | --- | --- |
|  | **N** | **%** | **N** | **%** |  |
| **Maternal perinatal characteristics** |  |  |  |  |  |
| **Family income quintile** |  |  |  |  | 0.967 |
| 1 | 707 | 19.6 | 620 | 19.1 |  |
| 2 | 721 | 20.1 | 647 | 19.9 |  |
| 3 | 705 | 19.6 | 649 | 19.9 |  |
| 4 | 761 | 21.2 | 698 | 21.4 |  |
| 5 | 700 | 19.5 | 641 | 19.7 |  |
| **Maternal age at birth** |  |  |  |  | 0.929 |
| < 18 | 329 | 9.2 | 303 | 9.3 |  |
| 18-21 | 720 | 20.0 | 632 | 19.4 |  |
| 22–34 | 2040 | 56.8 | 1864 | 57.3 |  |
| > 34 | 504 | 14.0 | 455 | 14.0 |  |
| **Maternal education at birth (in years)** |  |  |  |  | 0.911 |
| 0–4 | 521 | 14.7 | 457 | 14.2 |  |
| 5–8 | 1459 | 41.0 | 1313 | 40.8 |  |
| 9–11 | 1232 | 34.6 | 1139 | 35.3 |  |
| ≥ 12 | 344 | 9.7 | 314 | 9.7 |  |
| **Maternal religion** |  |  |  |  | 0.980 |
| No | 1732 | 53.5 | 1625 | 53.4 |  |
| Yes | 1506 | 46.5 | 1416 | 46.6 |  |
| **Participant characteristics** |  |  |  |  |  |
| **Sex** |  |  |  |  | 0.514 |
| Male | 1849 | 48.6 | 1648 | 50.6 |  |
| Female | 1746 | 51.4 | 1607 | 49.4 |  |
| **Gender** |  |  |  |  | 0.999 |
| Man | 1631 | 50.2 | 1627 | 50.2 |  |
| Woman | 1564 | 48.1 | 1555 | 48.0 |  |
| Both | 34 | 1.0 | 34 | 1.1 |  |
| Neither | 22 | 0.7 | 22 | 0.7 |  |
| **Race** |  |  |  |  | 0.895 |
| White | 2339 | 67.9 | 2185 | 68.1 |  |
| Mixed, Black, or other | 1105 | 32.1 | 1024 | 31.9 |  |
| **Nutritional status** |  |  |  |  | 0.999 |
| Underweight | 79 | 2.6 | 76 | 2.5 |  |
| Normal weight | 1939 | 63.1 | 1889 | 63.2 |  |
| Overweight | 577 | 18.8 | 565 | 18.9 |  |
| Obese | 475 | 15.5 | 462 | 15.4 |  |
| **History of smoking** |  |  |  |  | 0.940 |
| No | 2848 | 87.4 | 2830 | 87.5 |  |
| Yes | 411 | 12.6 | 405 | 12.5 |  |
| **Current smoker** |  |  |  |  | 0.929 |
| No | 3078 | 91.7 | 2965 | 91.8 |  |
| Yes | 279 | 8.3 | 266 | 8.2 |  |
| **History of drinking** |  |  |  |  | 0.620 |
| No | 479 | 14.2 | 447 | 13.8 |  |
| Yes | 2898 | 85.8 | 2802 | 86.2 |  |
| **Current alcohol use** |  |  |  |  | 0.999 |
| Low-risk consumption | 1314 | 65.4 | 1307 | 65.5 |  |
| Risky consumption | 575 | 28.6 | 571 | 28.6 |  |
| Harmful or high-risk use | 83 | 4.1 | 81 | 4.1 |  |
| Possible dependence | 37 | 1.8 | 36 | 1.8 |  |
| **Current drug use** |  |  |  |  | 0.999 |
| Never used | 2115 | 66.6 | 2099 | 64.7 |  |
| Tried it once | 440 | 13.5 | 438 | 13.5 |  |
| Used, but no longer uses | 286 | 8.7 | 284 | 8.7 |  |
| Occasional user | 267 | 8.2 | 264 | 8.1 |  |
| Uses only on weekends | 47 | 1.4 | 46 | 1.4 |  |
| Uses daily or almost daily | 117 | 3.6 | 117 | 3.6 |  |
| **Sexual orientation** |  |  |  |  | 0.999 |
| Heterosexual | 2391 | 74.9 | 2386 | 75.0 |  |
| Homosexual | 99 | 3.1 | 99 | 3.1 |  |
| Bisexual | 503 | 15.8 | 500 | 15.7 |  |
| Pansexual | 105 | 3.3 | 105 | 3.3 |  |
| Other | 93 | 2.9 | 92 | 2.9 |  |
| **Immunized for human papillomavirus** |  |  |  |  | 0.999 |
| No | 892 | 27.7 | 889 | 27.7 |  |
| Yes | 1622 | 50.4 | 1617 | 50.4 |  |
| Unknown | 705 | 21.9 | 703 | 21.9 |  |
